# Colchicine Protects against Ethanol-Induced Senescence and Senescence-Associated Secretory Phenotype in Endothelial Cells

**DOI:** 10.3390/antiox12040960

**Published:** 2023-04-19

**Authors:** Huakang Zhou, Dilaware Khan, Norbert Gerdes, Carsten Hagenbeck, Majeed Rana, Jan Frederick Cornelius, Sajjad Muhammad

**Affiliations:** 1 Department of Neurosurgery, Medical Faculty and University Hospital Düsseldorf, Heinrich-Heine-University Düsseldorf, 40225 Düsseldorf, Germany; 2 Division of Cardiology, Pulmonology and Vascular Medicine, University Hospital and Medical Faculty, Heinrich-Heine-University, 40225 Düsseldorf, Germany; 3 Clinic for Gynecology and Obstetrics, University Clinic, 40225 Düsseldorf, Germany; 4 Department of Oral, Maxillofacial and Facial Plastic Surgery, University Hospital Düsseldorf, Moorenstrasse 5, 40225 Düsseldorf, Germany; 5 Department of Neurosurgery, University Hospital Helsinki, Topeliuksenkatu 5, 00260 Helsinki, Finland

**Keywords:** ethanol, HUVECs, cellular senescence, SASP, inflammation, colchicine, NFκ-B, MAPKs

## Abstract

Inflammaging is a potential risk factor for cardiovascular diseases. It results in the development of thrombosis and atherosclerosis. The accumulation of senescent cells in vessels causes vascular inflammaging and contributes to plaque formation and rupture. In addition to being an acquired risk factor for cardiovascular diseases, ethanol can induce inflammation and senescence, both of which have been implicated in cardiovascular diseases. In the current study, we used colchicine to abate the cellular damaging effects of ethanol on endothelial cells. Colchicine prevented senescence and averted oxidative stress in endothelial cells exposed to ethanol. It lowered the relative protein expression of aging and senescence marker P21 and restored expression of the DNA repair proteins KU70/KU80. Colchicine inhibited the activation of nuclear factor kappa B (NFκ-B) and mitogen activated protein kinases (MAPKs) in ethanol-treated endothelial cells. It reduced ethanol-induced senescence-associated secretory phenotype. In summary, we show that colchicine ameliorated the ethanol-caused molecular events, resulting in attenuated senescence and senescence-associated secretory phenotype in endothelial cells.

## 1. Introduction

Inflammaging develops in older individuals and is characterized by elevated levels of pro-inflammatory markers in the serum and different tissues of healthy individuals [1]. Inflammaging is considered a causal risk factor for cardiovascular diseases [1,2]. Throughout life, senescent cells accumulate in vascular tissue, resulting in the development of inflammaging, which consequently leads to cardiovascular diseases [1,2,3,4]. Senescent cells have been observed in atherosclerotic tissue, and by eliminating senescent cells, the progression of atherosclerosis could be prevented [1,2,5]. The abolition of senescent cells has been shown to increase lifespan and provide protection against cardiovascular and age-related diseases in experimental animal studies [3,6].

Primary cells grow to a certain limit, after which they stop proliferating and reach growth arrest, a phenomenon termed the Hayflick limit [4,6]. These cells are labeled replicative senescent [4,6,7]. The senescent cells acquire a pro-inflammatory phenotype, called senescence-associated secretory phenotype (SASP) [1,3,8]. These cells increase the expression of cytokines, such as interleukin-1β (IL-β), IL-6, and tumor necrosis factor-α (TNF-α); chemokines, such as monocyte chemoattractant protein-1 (MCP-1) and IL-8; cell adhesion molecules, such as endothelial selectin (E-selectin), intercellular adhesion molecule-1 (ICAM-1), and vascular cell adhesion molecule-1 (VCAM-1); and matrix metalloproteinase (MMP) such as MMP-2 [1,3]. The SASP-associated molecules have been implicated in several cardiovascular diseases, including atherosclerosis, stroke, and myocardial infarction, and aneurysm formation and rupture [9,10,11,12,13,14,15]. The lack or inhibition of these molecules has been shown to reduce atherosclerosis formation [11,12,13,14] and decrease aneurysm formation and rupture [9,10,15] in different animal models. The expression of SASP-associated molecules is regulated by P38 via nuclear factor kappa B (NF-κB) transcriptional activity [3,16,17]. Previous studies have shown that inhibiting the activation of P38 and NF-κB delayed cellular senescence and attenuated SASP [18,19,20].

In addition to replicative senescence, other factors such as oxidative stress, DNA damage, oncogene activation or inactivation, epigenetic alterations, mitochondrial dysfunction, and exposure to damage-associated molecular patterns (DAMPs) released by stressed cells can also induce senescence in cells [1,6,7], contributing to hypertension, arterial stiffness, and atherosclerosis, and thereupon leading to cardiovascular diseases [2,4,5,21]. In addition to age, smoking, alcohol abuse, and hypertension are potential acquired risk factors for cardiovascular diseases. Previously, ethanol has been shown to induce senescence in endothelial [22] and other cell types [23,24], increase the expression of SASP molecules [22], and activate NF-κB and mitogen-activated protein kinases (MAPKs) [23,25,26]. Colchicine is an alkaloid known to impede inflammation and extenuate the expression of pro-inflammatory molecules. Colchicine dampens inflammation and attenuates extracellular remodeling by inhibiting endothelial dysfunction, platelet activation, and platelet aggregation; inhibiting the interaction between endothelial cells and platelets, inflammatory cells and endothelial cells, and platelets and inflammatory cells; and blocking NF-κB activation, resulting in the reduced expression of inflammatory and extracellular remodeling molecules [27]. Colchicine has been used to treat gout flares, familial mediterranean fever, calcium pyrophosphate disease, Adamantiades–Behcet’s syndrome, and pericarditis [28,29]. The results from published trials have shown that colchicine has provided benefits against cardiovascular diseases [27,28].

Here, we show that colchicine can reduce ethanol-induced cellular senescence and SASP by inhibiting NFκ-B and MAPKs activation.

## 2. Methods

### 2.1. Cell Culture

In the current study, we used human umbilical vein endothelial cells (HUVECs) purchased from Promocell (Heidelberg, Germany). The endothelial cell medium (C-22010, Promocell, Heidelberg, Germany) was added to the endothelial cell growth factors (C-39215, Promocell, Heidelberg, Germany) and used to maintain HUVECs. The cells were kept at 37 degrees Celsius (°C) in a 95% humidified atmosphere containing 5% CO_2_. After thawing, the cells were seeded in a T75 cell culture flask. The cells were passaged when they reached 90% confluency. For passaging, the cells were trypsinized at 37 °C for 4 min. A total of 5000 cells/cm^2^ were seeded in 10 cm culture plates for protein analysis and 6-well plates for mRNA analysis. For all experiments, endothelial cells at passage 7 were treated with endothelial cell medium containing either 400 millimolar (mM) ethanol (EtOH), 50 nanomolar (nM) colchicine, or 400 mM ethanol combined with 50 nM colchicine. The endothelial cells were treated with a higher concentration of ethanol (400 mM) to induce senescence and SASP over a short period. Controls were treated with endothelial cell medium only. For all experiments, the endothelial cells were treated with different conditions for 24 h, with the exception of immunofluorescence staining, for which the duration of treatment was 2 h. Colchicine was purchased from Sigma-Aldrich, St. Louis, MO, USA (C3915).

### 2.2. β-Gal Staining

β-Galactosidase (β-Gal) Reporter Gene Staining Kit (Sigma-Aldrich, St. Louis, MO, USA) was used for the detection of β-Gal expression. The manufacturer’s instructions were followed to stain the endothelial cells treated for 24 h with different conditions, as described in the Section 2.1. The fixation buffer provided with β-Galactosidase Reporter Gene Staining Kit was used to fix the treated cells. The fixed cells were incubated with the freshly prepared staining solution at 37 °C for 7 h. After that, the staining solution was removed, and the 70% glycerol solution was used to overlay the cells for storage at 4 °C. Using optical microscope, the images were captured. To count the stained cells, ImageJ 1.53c (National Institute of Health, Bethesda, MD, USA) was used. For the experiment, biological triplicates were used.

### 2.3. Immunofluorescence Staining

Immunofluorescence staining was performed as previously described [22]. After three washing steps with phosphate-buffered saline (PBS) (Thermo Fisher, Waltham, MA, USA), the endothelial cells were fixed with 4% paraformaldehyde (Thermo Fisher, Waltham, MA, USA) for 15 min. For permeabilization, the cells were incubated with 0.2% Triton™ X-100 (Sigma-Aldrich, St. Louis, MO, USA) for 10 min. Subsequently, the cells were treated with 5% bovine serum albumin (BSA) (VWR, Langenfeld, Germany) blocking solution for 1 h at room temperature (RT). After that, the cells were incubated with primary antibody 8-Hydroxydesoxyguanosin (8-OHDG) (1:500, Cat. No.: BSS-BS-1278R, BIOSS, Woburn, MA, USA) at 4 °C overnight. On the following day, the cells were washed three times with PBS and incubated with the secondary antibody (1:1000, Alexa Fluor 488, excitation 495 nm, emission 519 nm, Cat. No.: A48269, Thermo Fisher, Waltham, MA, USA) for 1 h at RT. SlowFade^®^ Gold Antifade Mountant with DAPI (Cat. No.: S36938, Thermo Fisher, Waltham, MA, USA) was used for nuclear staining. The images were captured at 20× magnification. The images were analyzed with ImageJ.

### 2.4. Western Blot (WB)

For protein analysis, the endothelial cells were treated with different conditions, as described in the Section 2.1 for 24 h. Radioimmunoprecipitation assay buffer was used for total protein extraction. DC protein Assay Kit (500-0116, Bio-Rad, Hercules, CA, USA) was used according to the manufacturer’s instructions to measure total protein concentration with the Paradigm micro-plate reader (Beckman Coulter, Krefeld, Germany). An amount of 25 microgram (µg) total protein in reducing conditions was loaded on 12% sodium dodecyl sulfate-polyacrylamide gel. The running conditions were 60 Volts for 20 min, followed by 110 Volts for 30–60 min. The polyvinylidene difluoride membranes were used for transfer at 250 milliampere for 120 min. The membranes were blocked with 5% BSA (0.05% tris buffered saline with tween (TBST)) for 1 h. Subsequently, membranes were incubated overnight at 4 °C with primary antibodies (Supplementary Table S1) and diluted in 5% BSA (0.05% TBST) on a shaking platform. On the next day, membranes were washed three times with TBST for 10 min. The membranes were incubated with secondary antibodies (Supplementary Table S1) at RT for 1 h. α-Tubulin was used as a loading control for all proteins except phosphorylated c-Jun N-terminal kinase (p-JNK), for which β-actin was used as the loading control. α-tubulin and β-actin were probed on different membranes. The probed WB membranes were scanned using Odyssey CLx Imaging system (LI-COR Biosciences, Lincoln, NE, USA). The untrimmed images of membranes have been provided in the Supplementary Material. ImageJ was used to calculate densitometry.

### 2.5. Quantitative Polymerase Chain Reaction (qPCR)

qPCR was performed as previously described [22]. Tri Reagent (T9424, Sigma-Aldrich, St. Louis, MO, USA) was used to extract total RNA. M-MLV Reverse Transcriptase kit (M1701, Promega, Walldorf, Germany) mixed with RiboLock RNase Inhibitor (EO0384, Thermo Fisher, Waltham, MA, USA) and Random Hexamer Primers (48190011, Thermo Fisher, Waltham, MA, USA) was used to reverse transcribe 1.2 µg total RNA. AceQ SYBR qPCR Master Mix (Q111-03, Vayzme, Nanjing, China) was used to perform qPCR on BIO-Rad CFX Connect Real-Time PCR System. The primer sequences are provided in Supplementary Table S2. The qPCR protocol was an initial denaturation of 95 °C for 8 min, followed by 45 cycles of 95 °C for 15 s, 58.9 °C for 30 s, and 72 °C for 30 s, which was then followed by a melting curve. β-actin was used for normalizing to calculate relative mRNA expression. To quantify relative mRNA expression, comparative C_T_ method was used [30]. For the experiment, biological triplicates were used. For each biological replicate, we used three technical triplicates.

### 2.6. Statistical Analysis

For statistical analysis, we performed one-way analysis of variance (ANOVA) followed by Tukey’s test. The level of significance was set to less than 0.05 (* *p* < 0.05).

## 3. Results

### 3.1. Colchicine Inhibited Ethanol-Induced Senescence

**Figure 1:**
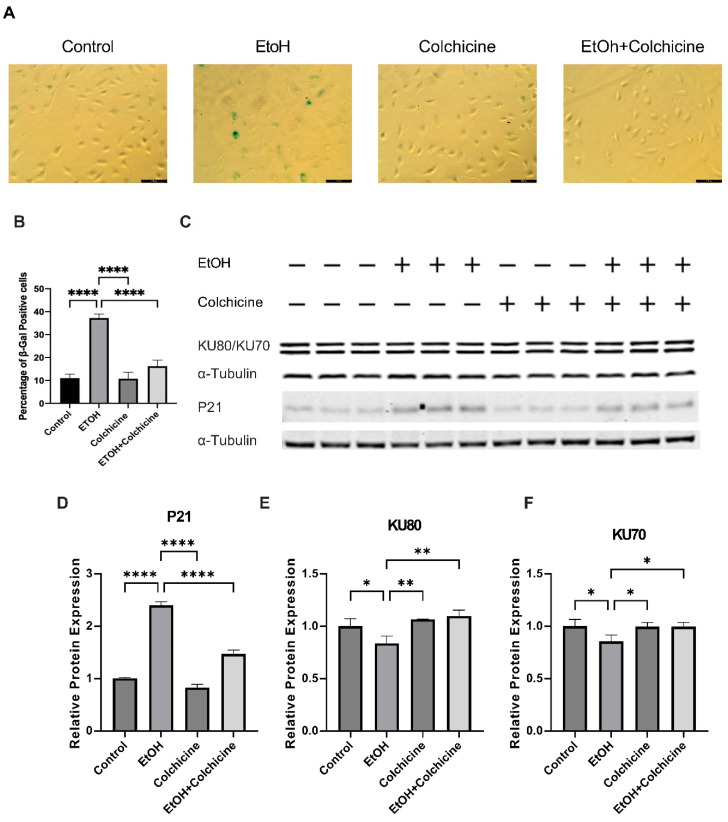
Colchicine inhibited endothelial senescence and restored the relative protein expression of aging-associated biomarkers. (**A**) Senescence in endothelial cells after 24 h of treatment with different conditions. (**B**) Colchicine subdued ethanol (EtOH)-induced senescence in endothelial cells. (**C**) Western blot showing protein expression of P21, KU80, and KU70. (**D**) Colchicine attenuated the relative protein expression of P21 in endothelial cells after ethanol exposure. (**E**,**F**) Colchicine recovered the relative protein expression of KU70 and KU80 in endothelial cells treated with ethanol. α-Tubulin was used as a loading control. Data are the mean of independent biological triplicates. The data was analyzed by performing one-way ANOVA followed by Tukey’s test. Scale bar = 100 µm; error bars represent the SD (**** *p* < 0.0001, ** *p* < 0.01, and * *p* < 0.05).

Ethanol is a known risk factor for cardiovascular diseases. We have already reported that ethanol increases cellular senescence in endothelial cells [22]. To investigate the effects of colchicine, we treated endothelial cells with either an endothelial cell medium alone (control) or an endothelial cell medium combined with either 400 mM ethanol, 50 nM colchicine, or 400 mM ethanol combined with 50 nM colchicine. After 24 h, we performed β-gal staining. Colchicine inhibited ethanol-induced cellular senescence in endothelial cells and lowered the percentage of β-gal positive cells (control = 11.01 ± 1.772%, EtOH = 37.21 ± 1.761%, colchicine = 10.76 ± 2.820%, EtOH + colchicine = 16.22 ± 2.630%, **** *p* < 0.0001, *n* = 3; Figure 1A,B). Next, we investigated the effect of colchicine on aging-associated biomarkers. Colchicine attenuated the relative protein expression of aging-associated biomarker P21 (control = 1.003 ± 0.018, EtOH = 2.398 ± 0.068, colchicine = 0.830 ± 0.060, EtOH + colchicine = 1.468 ± 0.075, **** *p* < 0.0001, *n* = 3; Figure 1C,D). P21 is a cyclin-dependent kinase (CDK) inhibitor, which establishes indefinite cell cycle arrest via the inhibition of CDK-2/4 [21]. The CDK-2/4 inhibition results in the active hypo-phosphorylated form of retinoblastoma protein, which, in turn, mediates cell cycle arrest and senescence phenotypes [21]. Colchicine also recovered the relative protein expression of DNA repair proteins KU70 (control = 1.000 ± 0.066, EtOH = 0.856 ± 0.060, colchicine = 0.995 ± 0.040, EtOH + colchicine = 0.995 ± 0.040, * *p* < 0.05, ** *p* < 0.01, *n* = 3; Figure 1C,E) and KU80 (control = 1.000 ± 0.072, EtOH = 0.838 ± 0.069, colchicine = 1.065 ± 0.004, EtOH + colchicine = 1.097 ± 0.058, * *p* < 0.05, *n* = 3, Figure 1C,F). The reduced expression of KU70 and KU80 has been observed in senescent cells compared to young cells [31]. KU70 and KU80 form a heterodimer and repair DNA double-strand breaks via a nonhomologous end-joining pathway [32]. KU70 and KU80 maintain telomere length, and the inactivation of KU70 and KU80 results in the shortening of telomere length in various primary cell types [33], leading to cellular senescence. Furthermore, KU80 has been shown to hinder oxidative stress-induced DNA damage [34].

Colchicine inhibited ethanol-induced endothelial senescence, ameliorated the relative protein expression of aging-associated biomarker P21, and restored the relative protein expression of the DNA repair proteins KU70 and KU80.

### 3.2. Colchicine Averted Ethanol-Induced Oxidative Stress in Endothelial Cells

**Figure 2:**
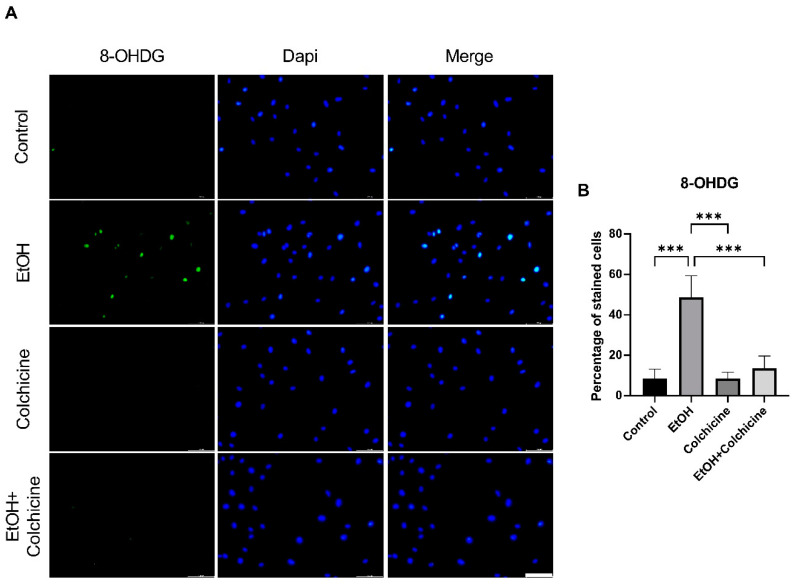
Colchicine restrained ethanol-induced oxidative stress in endothelial cells. (**A**) Immunofluorescence staining for oxidative stress marker 8-Hydroxydesoxyguanosin (8-OHDG) in endothelial cells 2 h after treatment with ethanol, colchicine, and ethanol combined with colchicine. Endothelial medium alone was used for untreated control. (**B**) Colchicine averted the expression of 8-OHDG in ethanol-treated cells. The experiment was performed with independent biological triplicates. The data was analyzed by performing one-way ANOVA followed by Tukey’s test. Scale bar = 100 µm; error bars represent the SD (*** *p* < 0.001).

The metabolism of ethanol produces reactive oxygen species (ROS) and reactive nitrogen species, resulting in increased oxidative stress [35]. Colchicine inhibited ethanol-induced oxidative stress in endothelial cells and lowered the percentage of oxidative stress-associated biomarker 8-OHDG positive cells (control = 8.569 ± 4.573%, EtOH = 48.62 ± 10.74%, colchicine = 8.553 ± 3.115%, EtOH + colchicine = 13.60 ± 6.001%, *** *p* = 0.001, *n* = 3; Figure 2). Colchicine obviated oxidative stress in ethanol-treated endothelial cells, which is in accordance with the previously reported findings [36,37].

### 3.3. Colchicine Suppressed the Activation of NF-κB and MAPKs in Ethanol-Treated Endothelial Cells

**Figure 3:**
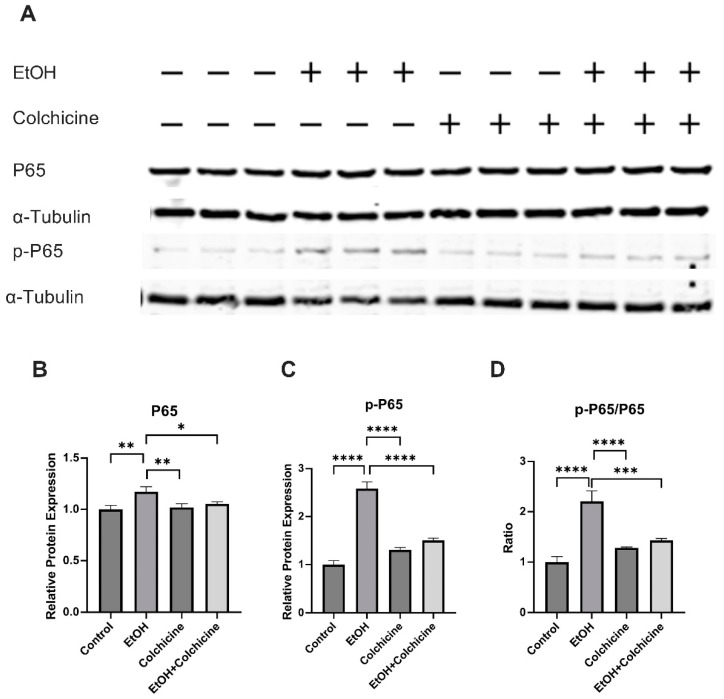
Colchicine inhibited nuclear factor kappa B (NF-κB) activation: (**A**) Western blots for proteins of P65 and p-P65. Colchicine obviated the relative protein expression of (**B**) P65 and (**C**) p-P65 in ethanol-treated endothelial cells. (**D**) The ratio of p-P65/P65 was significantly decreased in ethanol-treated endothelial cells exposed to colchicine. α-Tubulin was used as loading control. Biological triplicates were used for the experiment. Data was analyzed by performing one-way ANOVA followed by Tukey’s test. Error bars represent the SD (**** *p* < 0.0001, *** *p* < 0.001, ** *p* < 0.01, and * *p* < 0.05).

To investigate the pathways of interest, we performed a protein analysis. NFκ-B has been suggested to play an important role in inflammation, aging, and cellular senescence [8,19]. Our protein analysis showed that colchicine inhibited the ethanol-induced relative protein expression of NF-κB subunit P65 (control = 1.000 ± 0.038, EtOH = 1.172 ± 0.049, colchicine = 1.019 ± 0.034, EtOH + colchicine = 1.053 ± 0.019, * *p* < 0.05, ** *p* < 0.01, *n* = 3; Figure 3A,B). Colchicine also abated the NF-κB activation, and reduced the relative protein expression of NF-κB subunit p-P65 (control = 0.9999 ± 0.083, EtOH = 2.580 ± 0.1417, colchicine = 1.310 ± 0.0488, EtOH + colchicine = 1.506 ± 0.0444, **** *p* < 0.0001, *n* = 3; Figure 3A,C) and *p*-P65/P65 (control = 1.002 ± 0.107, EtOH = 2.208 ± 0.208, colchicine = 1.285 ± 0.014, EtOH + colchicine = 1.431 ± 0.037, *** *p* < 0.001, **** *p* < 0.0001, *n* = 3; Figure 3A,D) was significantly reduced after colchicine treatment in ethanol-treated endothelial cells.

**Figure 4:**
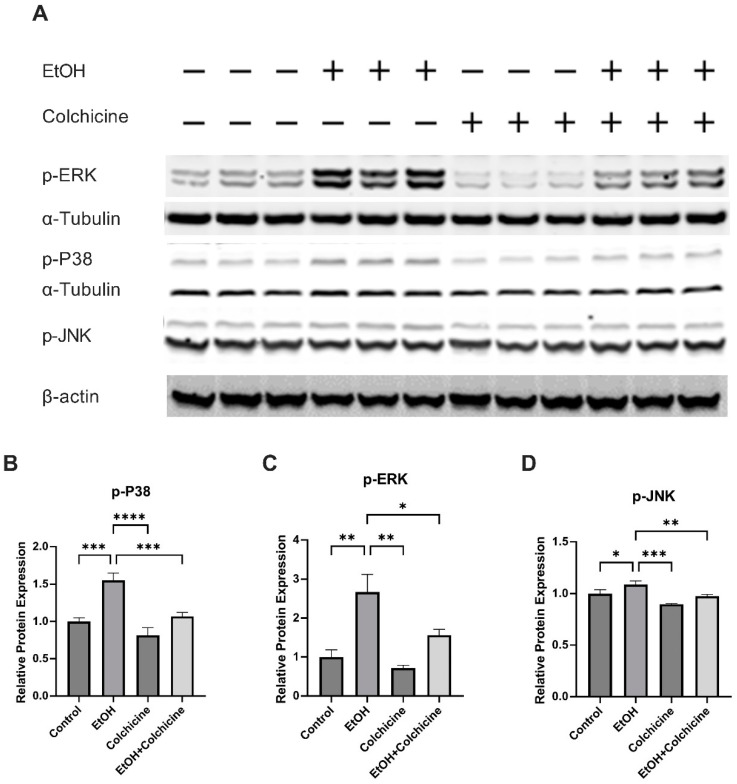
Colchicine inhibited mitogen activated protein kinases (MAPKs) activation: (**A**) Western blots for proteins of p-P38, phosphorylated extracellular signal-regulated protein kinase (p-ERK), and phosphorylated c-Jun N-terminal kinase (p-JNK). Colchicine averted relative protein expression of (**B**) p-P38, (**C**) p-ERK, and (**D**) p-JNK in ethanol-treated endothelial cells. α-Tubulin was used as a loading control for all proteins expression except p-JNK. β-actin was used as a loading control for only p-JNK on a separate membrane. The experiment was performed with independent biological triplicates. The data was analyzed by performing one-way ANOVA followed by Tukey’s test. Error bars represent the SD (**** *p* < 0.0001, *** *p* < 0.001, ** *p* < 0.01, and * *p* < 0.05).

It has previously been reported that ethanol activates MAPKs [25] and MAPKs have been implicated in cellular senescence. The protein analysis showed that colchicine impeded the ethanol-induced activation of MAPKs: the relative protein expression of p-P38 (control = 0.9988 ± 0.050, EtOH = 1.553 ± 0.098, colchicine = 0.814 ± 0.103, EtOH + colchicine = 1.070 ± 0.051, *** *p* <= 0.001, **** *p* < 0.0001, *n* = 3; Figure 4A,B), phosphorylated extracellular signal-regulated protein kinase (p-ERK) (control = 1.001 ± 0.1834, ETOH = 2.667 ± 0.4533, colchicine = 0.7198 ± 0.0671, EtOH + colchicine = 1.566 ± 0.1465, ** *p* < 0.01, *** *p* < 0.001, **** *p* < 0.0001, *n* = 3; Figure 4A,C), and p-JNK (control = 1.000 ± 0.039, EtOH = 1.088 ± 0.035, colchicine = 0.899 ± 0.005, EtOH + colchicine = 0.976 ± 0.017, * *p* < 0.05, ** *p* < 0.01, *** *p* < 0.001, *n* = 3; Figure 4A,D).

### 3.4. Colchicine Ameliorated Ethanol-Induced SASP in Endothelial Cells

**Figure 5:**
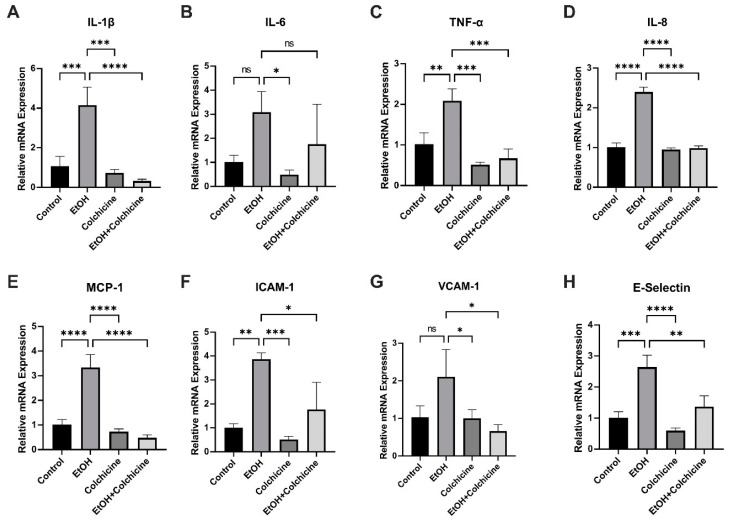
Colchicine attenuated the relative mRNA expression of SASP-associated inflammatory markers. Colchicine reduced the relative mRNA expression of (**A**) interleukin (IL)-1β, (**C**) tumor necrosis factor-α (TNF-α), (**D**) IL-8, (**E**) monocyte chemoattractant protein-1 (MCP-1), (**F**) intercellular adhesion molecule-1 (ICAM-1), and (**H**) endothelial selectin (E-Selectin) in ethanol-treated endothelial cells. Ethanol did not significantly increase the relative mRNA expression of (**B**) IL-6 or (**G**) vascular cell adhesion molecule-1 (VCAM-1). Colchicine significantly lowered the relative mRNA expression of VCAM-1 in ethanol-treated endothelial cells. β-actin was used as a loading control. qPCR data are the mean of three independent technical replicates. The data was analyzed by performing one-way ANOVA followed by Tukey’s test. Error bars represent the SD (**** *p* < 0.0001, *** *p* < 0.001, ** *p* < 0.01, and * *p* < 0.05), ns: not significant.

Senescent cells acquire SASP, which is characterized by the increased expression and release of inflammatory cytokines, chemokines, proteases, and growth factors [1,3,8]. Because colchicine inhibited senescence and senescence-associated pathways in ethanol-treated endothelial cells (Figure 1, Figure 3 and Figure 4), we investigated the relative mRNA expression of SASP-associated cytokines, chemokines, and cell adhesion molecules. Colchicine curtailed the relative mRNA expression of the cytokines IL-1β (control = 1.067 ± 0.4931, EtOH = 4.146 ± 0.9146, colchicine = 0.7295 ± 0.1732, EtOH + colchicine = 0.3203 ± 0.09465, *** *p* < 0.001, **** *p* < 0.0001, *n* = 3; Figure 5A) and TNF-α (control = 1.023 ± 0.2783, EtOH = 2.089 ± 0.2934, colchicine = 0.5135 ± 0.0654, EtOH + colchicine = 0.6711 ± 0.2300, ** *p* < 0.01, *** *p* < 0.001, *n* = 3; Figure 5C). It also reduced the relative mRNA expression of the chemokines IL-8 (control = 1.004 ± 0.1091, EtOH = 2.398 ± 0.1235, colchicine = 0.9490 ± 0.0410, EtOH + colchicine = 0.9802 ± 0.0586, **** *p* < 0.0001, *n* = 3; Figure 5D) and MCP-1 (control = 1.013 ± 0.2085, EtOH = 3.341 ± 0.5194, colchicine = 0.7364 ± 0.1053, EtOH + colchicine = 0.4826 ± 0.1187, **** *p* < 0.0001, *n* = 3; Figure 5E). Finally, it decreased the relative mRNA expression of the cell adhesion molecules ICAM-1 (control = 1.009 ± 0.1649, EtOH = 3.871 ± 0.2693, colchicine = 0.5126 ± 0.1318, EtOH + colchicine = 1.767 ± 1.133, * *p* < 0.05, ** *p* < 0.01, *** *p* < 0.001, *n* = 3; Figure 5F) and E-Selectin (control = 1.012 ± 0.1944, EtOH = 2.645 ± 0.3812, colchicine = 0.5963 ± 0.0850, EtOH + colchicine = 1.366 ± 0.3571, ** *p* < 0.01 *** *p* < 0.001, **** *p* < 0.0001, *n* = 3; Figure 5H). Ethanol did not significantly increase the relative mRNA expression of IL-6 (control = 1.024 ± 0.2673, EtOH = 3.092 ± 0.8467, colchicine = 0.4912 ± 0.1902, EtOH + colchicine = 1.756 ± 1.660, * *p* < 0.05, *n* = 3; Figure 5B) or VCAM-1 (control = 1.028 ± 0.3069, EtOH = 2.108 ± 0.7257, colchicine = 1.004 ± 0.2258, EtOH + colchicine = 0.6622 ± 0.1701, * *p* < 0.05, *n* = 3; Figure 5G). In conclusion, colchicine reduced the relative mRNA expression of SASP-associated cytokines, chemokines, and cell adhesion molecules in endothelial cells exposed to ethanol.

### 3.5. Colchicine Mitigated Ethanol-Induced Relative mRNA and Relative Protein Expression of MMP-2

**Figure 6:**
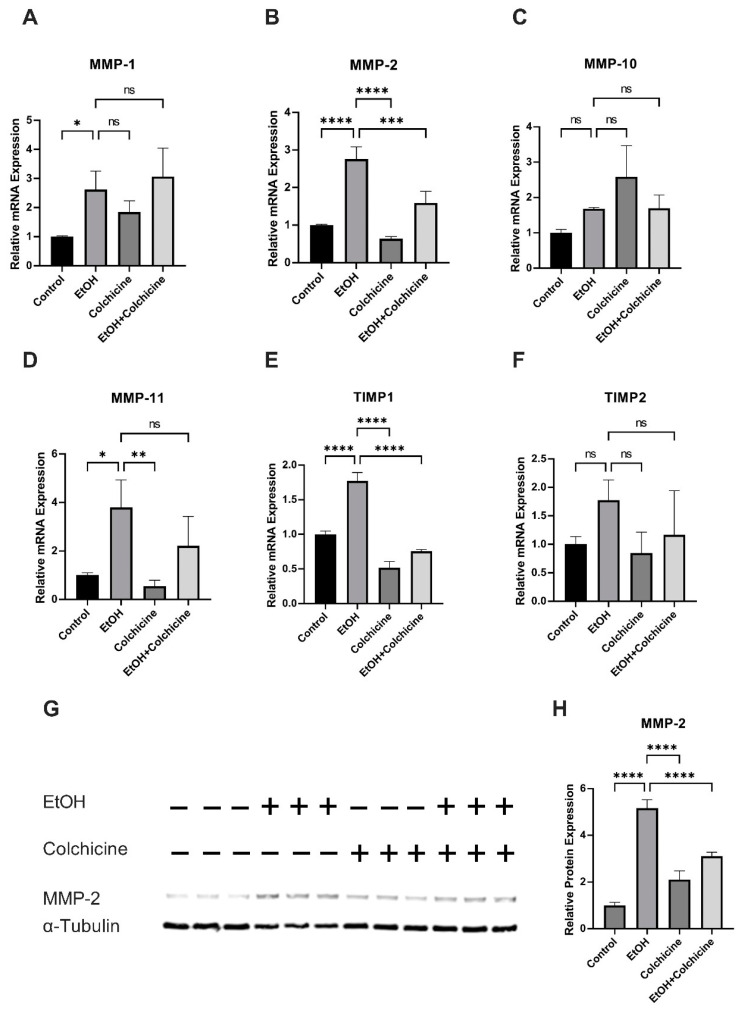
Colchicine abated the relative mRNA and relative protein expression of matrix metalloproteinase (MMP)2. Colchicine did not ameliorate the relative mRNA expression of (**A**) MMP1 or (**D**) MMP-11. Ethanol and colchicine did not alter the relative mRNA expression of (**C**) MMP-10 or (**F**) tissue inhibitor of metalloproteinase (TIMP)2. Colchicine attenuated relative mRNA expression of (**E**) TIMP1 and (**B**) MMP-2 and relative protein expression of (**G**,**H**) MMP-2 in ethanol-treated cells. β-actin for qPCR and α-tubulin for WB were used as a loading control. qPCR data are the mean of three independent technical replicates and WB data are the mean of the independent biological triplicates. The data was analyzed by performing one-way ANOVA followed by Tukey’s test. Error bars represent the SD (**** *p* < 0.0001, *** *p* < 0.001, ** *p* < 0.01, and * *p* < 0.05), ns: not significant.

Colchicine did not decrease the ethanol-induced relative mRNA expression of MMP-1 (control = 1.000 ± 0.0285, ETOH = 2.617 ± 0.6381, colchicine = 1.847 ± 0.3830, EtOH + colchicine = 3.069 ± 0.9726, * *p* < 0.05, *n* = 3; Figure 6A) or MMP-11 (control = 1.003 ± 0.0929, ETOH = 3.795 ± 1.136, colchicine = 0.5490 ± 0.2415, EtOH + colchicine = 2.214 ± 1.214, * *p* < 0.05, ** *p* < 0.01, *n* = 3; Figure 6D). Both ethanol and colchicine alone or in combination did not significantly affect the relative mRNA expression of MMP-10 (control = 1.003 ± 0.0932, EtOH = 1.686 ± 0.0314, colchicine = 2.585 ± 0.8815, EtOH + colchicine = 1.693 ± 0.3770, *n* = 3; Figure 6C) or TIMP2 (control = 1.004 ± 0.1296, ETOH = 1.775 ± 0.3543, colchicine = 0.8472 ± 0.3676, EtOH + colchicine = 1.165 ± 0.7765, *n* = 3; Figure 6F). Colchicine inhibited the ethanol-induced relative mRNA expression of TIMP1 (control = 1.001 ± 0.04815, EtOH = 1.773 ± 0.1194, colchicine = 0.5182 ± 0.0901, EtOH + colchicine = 0.7562 ± 0.02403, **** *p* < 0.0001, *n* = 3; Figure 6E) and MMP-2 (control = 1.000 ± 0.0212, EtOH = 2.761 ± 0.3196, colchicine = 0.6408 ± 0.0545, EtOH + colchicine = 1.591 ± 0.3091, *** *p* < 0.001, **** *p* < 0.0001, *n* = 3; Figure 6B). Colchicine also attenuated the relative protein expression of MMP-2 (control = 1.00 ± 0.127, EtOH = 5.161 ± 0.356, colchicine = 2.102 ± 0.381, EtOH + colchicine = 3.102 ± 0.177, **** *p* < 0.0001, *n* = 3; Figure 6G,H).

## 4. Discussion

We showed that colchicine averted cellular senescence and SASP in ethanol-treated endothelial cells. The pathway analysis showed that colchicine inhibited the activation of NF-κB, P38, ERK, and JNK pathways in endothelial cells exposed to ethanol. Ethanol is a potential risk factor for cardiovascular diseases. We, in addition to other researchers, have previously reported that alcohol causes cellular senescence [22,23]. Cellular senescence contributes to cardiovascular diseases via the increase in inflammaging in the vascular endothelium [1,2,3,4]. In the current study, we investigated the effects of colchicine on ethanol-treated endothelial cells.

The ethanol treatment induced cellular senescence in endothelial cells (Figure 1) [22,23,24]. Colchicine inhibited cellular senescence and attenuated oxidative stress in ethanol-treated endothelial cells (Figure 1 and Figure 2). Previously, colchicine has been shown to exert anti-oxidative effects by upregulating the expression of anti-oxidant enzymes such as CAT, GPx-1, and SOD2 in platelets [38]. Ethanol metabolism results in the formation of ROS, which leads to an increase in oxidative stress [35]. The over-accumulation of ROS causes DNA damage and triggers cellular senescence. Colchicine, by inhibiting ROS generation in vivo and in vitro [36,37,38], can decrease oxidative stress and DNA damage. DNA damage and oxidative stress can activate NF-κB and MAPKs [8,39]. In addition to this, ethanol can activate NF-κB and MAPKs via TLR4/Type I IL-1 receptor signaling [25]. The pathway analysis showed that ethanol activated NF-κB, P38, JNK, and ERK pathways in HUVECs (Figure 3 and Figure 4), which is in agreement with the previously reported findings [22,23,25,26]. These pathways modulate inflammation, contribute to cellular senescence [8,20,39,40,41], and increase the transcription and expression of cell cycle inhibitor protein P21 via different mechanisms [39,42,43,44,45,46]. The NF-κB activation enhanced the expression of cell cycle inhibitor P21 in response to DNA damage that was independent of the P53 pathway [42]. P38 increases P21 expression by enhancing the expression, stabilization, and promoter activity of P53 [43,44]. Moreover, P38 phosphorylates HuR, which, in turn, increases the cytoplasmic accumulation of HuR and the binding of HuR to P21, consequently improving the stability of P21 mRNA and, thus, enhancing P21 protein levels [45]. ERK1/2 promotes the transcription of P21 via the ELK1, SP1 and SMAD proteins [39,46]. The inhibition of NF-κB and MAPKs activation delayed cellular senescence [18,19,20], suggesting that inflammation can induce premature senescence in endothelial cells. Colchicine inhibited ethanol-induced senescence by attenuating oxidative stress, recovering the protein expression of KU70 and KU80, ameliorating P21 protein expression, and inhibiting the NF-κB, P38, ERK, and JNK pathways in endothelial cells.

Because Colchicine attenuated ethanol-induced senescence and inhibited the ethanol-induced activation of pro-inflammatory pathways, we investigated the effect of colchicine on SASP-associated cytokines (IL-1β, IL-6, and TNF-α), chemokines (IL-8 and MCP-1), and cell adhesion molecules (ICAM-1, VCAM-1, and E-Selectin) [1,3,8]. Colchicine inhibited the expression of these SASP-associated pro-inflammatory molecules (Figure 5) [38,47,48]. This pro-inflammatory response in senescent and dysfunctional endothelial cells is regulated by the NF-κB complex [3,8]. MAPKs have been suggested to be the upstream regulators of NF-κB [16]. P38 controls NF-κB activity in senescent cells and it induces SASP by increasing the mRNA expression of SASP molecules primarily via NF-κB transcriptional activity [3,16,17].

Senescent endothelial cells promote atherosclerosis and thrombosis by the increased expression and release of SASP-associated inflammatory factors and chemokines [3,8]. MCP-1, IL-8, and cell adhesion molecules E-selectin, VCAM-1, and ICAM-1 promote the extravasation of inflammatory cells from the blood stream across the endothelium [13,49,50,51]. These infiltrated inflammatory cells create a pro-inflammatory microenvironment, exacerbate inflammation, and lead to atherosclerotic plaque formation [50]. Colchicine reduced the adhesion of monocytes to HUVECs by inhibiting the expression of adhesion molecules VCAM-1 and ICAM-1 [38]. Colchicine decreased the recruitment of monocytes and neutrophils into the atherosclerotic plaque of mice aorta [47]. In addition to their role in the tissue infiltration of inflammatory cells, the SASP-associated molecules have the potential to activate the inflammatory cells [49,50]. MCP-1 activates immune cells, such as monocytes, and regulates the polarization of T-cells and the differentiation of monocytes into dendritic cells [50]. IL-1β and TNF-α released from senescent endothelial cells can activate inflammatory cells, neighboring endothelial and smooth muscle cells [52,53]. Both IL-1β and TNF-α have been shown to decrease collagen synthesis and increase the mRNA expression and activity of MMPs [54], which can consequently result in tissue remodeling [55,56]. The TNF-α-induced phenotype switch in smooth muscle cells can impair vasorelaxation [52]. TNF-α can cause endothelial cell apoptosis and smooth muscle cell proliferation and migration [53,57], leading to the initiation and progression of cardiovascular diseases. TNF-α increased the expression of E-selectin, VCAM-1, and ICAM-1 in HUVECs [58]. Colchicine reduced TNF-α, IL-1β, MCP-1, and ICAM-1 expression at mRNA and protein levels in vivo and in vitro [38,47,48]. Cerebral aneurysm formation and rupture were significantly reduced in MCP1-, TNF-α-, and TNF-α-R1 deficient mice [9,10,15]. The lack of MCP-1, MCP-1 receptor CCR2 inhibition, IL-1β deficiency, and TNF-α inhibition have been shown to decrease atherosclerosis formation [11,12,13,14]. The rupture of atherosclerotic plaque leads to thrombus formation. The aggregation, activation, and inflammatory response of platelets is known to play a key role in atherosclerosis and thrombus formation [59]. Colchicine blocked platelet–platelet aggregation in both whole blood and platelet-rich plasma, platelet activation (ROS generation), and procoagulant platelet formation [37,38,60]. The addition of colchicine in whole blood, in vitro, and in oral administration in healthy subjects, in vivo, decreased monocyte-platelet and neutrophil-platelet aggregation [60]. Colchicine has been shown to reduce NETs formation [61], which has been suggested to contribute to thrombosis by accumulating prothrombotic factors such as fibrinogen and von Willebrand factor, and by promoting platelet adhesion, activation, and aggregation [62]. In mice, colchicine inhibited carrageenan-induced thrombosis and ameliorated platelet activation [38]. The authors also showed that colchicine dampened human platelet activation by inhibiting the activation of AKT pathway, which subsequently blocked ERK1/2 activation [38]. These findings indicate that colchicine, by inhibiting senescence and curtailing SASP-induced sterile inflammation in the endothelium, can be potentially useful against cardiovascular diseases [27,28].

Previous studies have shown that senescent cells increase the expression of MMPs [1,3,8,21]. In addition, ethanol has been shown to increase the expression of MMPs in endothelial [22] and other cell types [63]. Alcohol consumption has been reported to increase serum levels of MMPs in alcohol abusers [64] and, in animal studies, alcohol elevated MMP expression in different tissues [65,66,67]. In the current study, ethanol increased the expression of MMPs (Figure 6). Colchicine inhibited the expression of MMP-2 at mRNA and protein levels (Figure 6). MMPs are known to contribute to cardiovascular diseases via tissue remodeling and scar formation, facilitating the migration and proliferation of smooth muscle cells, the infiltration of inflammatory cells such as monocytes and neutrophils into the endothelium, and promoting inflammation via their protease function on cytokines and chemokines [55,56]. These findings suggest that, by inhibiting MMP-2 mRNA and protein expression, colchicine can potentially suppress tissue remodeling and MMP-2-mediated inflammation in endothelial cells.

## 5. Conclusions

Alcohol is a potential risk factor for cardiovascular diseases. In the current study, we showed that ethanol induced premature senescence and SASP. Colchicine reduced ethanol-induced inflammaging in HUVECs possibly by inhibiting the activation of the NF-kB and MAPKs pathways. Thus, colchicine could be a potential pharmacological target for cardiovascular diseases.

## 6. Limitations

Our study has some limitations. The exposure of the endothelial cells to ethanol, for example, was acute and not chronic. The concentration of ethanol (400 mM) used was much higher and the ethanol evaporation from culture media was not prevented. This higher concentration of ethanol was used to induce senescence and SASP in endothelial cells over a short period. Moreover, the study was conducted using endothelial cells in vitro. In addition, we used HUVECs as endothelial cells. The data should be carefully interpreted.

## Data Availability

All data generated or analyzed during this study are included in this published article.

## Supplementary Materials

The following supporting information can be downloaded at: https://www.mdpi.com/article/10.3390/antiox12040960/s1, Supplementary Table S1. The list of antibodies used in the study. Supplementary Table S2. The list of primers.
