# Supplementary material for: Colchicine Protects against Ethanol-Induced Senescence and Senescence-Associated Secretory Phenotype in Endothelial Cells
Source: Antioxidants (Basel). 2023 Apr 19;12(4):960. doi: 10.3390/antiox12040960 (PMC10135532; doi:10.3390/antiox12040960)
Supplement: Supplementary file 1 [file antioxidants-12-00960-s001.zip › antioxidants-2310088-supplementary.pdf]

Table S1. Primary and Secondary antibodies

Antibody	MW(kDa)	Brand	Catalog Number	Concentration
P21	21	Cell Signaling	# 2947S	1:1,000
KU70	70	Cell Signaling	#4588S	1:1,000
KU80	86	Cell Signaling	#2753S	1:1,000
P65	65	Abcam	ab16502	1:1,000
p-P65	65	Cell Signaling	#3033S	1:1,000
MMP-2	70	Invitrogen	436000	2 µg/mL
β-actin	45	Cell Signaling	#4970S	1:1,000
α-Tubulin	50	Sigma Aldrich	#T9026	1:500
p-P38	43	Cell Signaling	#4511S	1:1,000
p-JNK	46,54	Cell Signaling	#9255S	1:2,000
p-ERK	42,44	Cell Signaling	#4370S	1:2,000
8-OHDG		BIOSS	BSS-BS-1278R	1:500
Alexa Fluor Plus 488 Excitation: 495 nm Emission: 519 nm		Thermo Fisher	A48269	1:1000
IRDye 800CW Goat-anti-Rabbit Antibody Excitation: 778 nm Emission: 795 nm		LiCor	# LI-COR 92632211	1:10,000
IRDye 680RD Donkey anti-Mouse IgG Secondary Antibody Excitation: 676 nm Emission: 694 nm		LiCor	926-68072	1:10,000

Table S2. Primer list

Target gene	Gene Accession Number	Sense 5' -3'	Antisense 5' -3'
ICAM-1	NM_000201	CACAGTCACCTATGGCAACGA	TGGCTTCGTCAGAATCACGTT
VCAM-1	NM_080682, NM_001199834, NM_001078	AGTGGTGGCCTCCTGAATGG	CTGTGTCTCCTGTCTCCGCT
IL-8	NM_001354840, NM_000584	TGCCAAGGAGTGCTAAAG	CTCCACAACCCTCTGCAC
MCP-1	NM_002982	CACCAATAGGAAGATCTCAGTGC	TGAGTGTTCAAGTCTTCGGAGTT
MMP1	NM_001145938, NM_002421	CAGAGATGAAGTCCGGTTTTTC	GGGGTATCCGTGTAGCACAT
MMP2	NM_001302510, NM_001302509, NM_001127891, NM_004530, NM_001302508	ATAACCTGGATGCCGTCGT	AGGCACCCTTGAAGAAGTAGC
MMP10	NM_002425	CACAGTTTGGCTCATGCCTA	TGCCATTCACATCATCTTGC
MMP11	NM_005940	CCGCAACCGACAGAAGAGG	ATCGCTCCATACCTTTAGGGC
TIMP1	NM_003254	TGGCTTCTGGCATCCTGTTGTTG	CGCTGGTATAAGGTGGTCTGGTTG
TIMP2	NM_003255	GAATCGGTGAGGTCCTGTCCTGA	CCTGCACACAAGCCCGGATAAA
IL-1β	NM_000576	AGATGATAAGCCCACTCTACAG	ACATTCAGCACAGGACTCTC
E-selectin	NM_000450	CAAGAAGAAGCTTGCCCTATG	ACTTGAGTCCACTGAAGCCA
TNF-α	NM_000594	CCCGAGTGACAAGCCTGTAG	GATGGCAGAGAGGAGGTTGAC
IL-6	NM_001371096 NM_001318095 XM_011515391 NM_000600	CTGCAGGACATGACAACTCATC	ATCTGAGGTGCCCATGCTAC
